# MicroRNAs as Plasma Biomarkers of Hepatocellular Carcinoma in Patients with Liver Cirrhosis—A Cross-Sectional Study

**DOI:** 10.3390/ijms25042414

**Published:** 2024-02-19

**Authors:** Robin Zenlander, Hugh Salter, Stefan Gilg, Gösta Eggertsen, Per Stål

**Affiliations:** 1 Department of Clinical Chemistry, Karolinska University Hospital, 141 86 Stockholm, Sweden; 2 Department of Laboratory Medicine, Karolinska Institutet, 141 52 Stockholm, Sweden; 3 Department of Medicine, Huddinge, Karolinska Institutet, 141 86 Stockholm, Swedenper.stal@ki.se (P.S.); 4 Division of Hepatology, Department of Upper GI Diseases, Karolinska University Hospital, 141 86 Stockholm, Sweden

**Keywords:** HCC, surveillance, circulating biomarkers, miRNA, proteins

## Abstract

Ultrasound screening for hepatocellular carcinoma (HCC) in patients with liver cirrhosis has a poor sensitivity for small tumors. Circulating microRNAs (miRNAs) have been explored as HCC biomarkers, but results are diverging. Here, we evaluate if miRNAs up-regulated in HCC tissue can be detected in plasma and used as screening biomarkers for HCC. In this cross-sectional study, plasma, HCC tissue and surrounding non-tumorous liver tissue were collected from liver resections. Tissue miRNAs were identified and quantitated by RNA-sequencing analysis, and the fold-changes between HCC and surrounding liver tissue were calculated. The miRNAs up-regulated in HCCs were then re-analyzed in plasma from the same patients, and the miRNAs with the highest plasma levels were subsequently measured in plasma from an independent cohort of patients with cirrhosis or HCC. In tissues from 84 resected patients, RNA-sequencing detected 197 differentially expressed miRNAs, 40 of which had a raw count above 200 and were analyzed in plasma from the same cohort. Thirty-one miRNAs were selected for further analysis in 200 patients with HCC or cirrhosis. Of these, eleven miRNAs were significantly increased in HCC as compared to cirrhosis patients. Only miR-93-5p and miR-151a-3p were significantly associated with HCC, with an AUC of 0.662. In comparison, alpha-fetoprotein and des-gamma-carboxy prothrombin yielded an AUC of 0.816, which increased to 0.832 if miR-93-5p and miR-151a-3p were added. When including sex and age, the addition of miR-93-5p and miR-151a-3p did not further improve the AUC (from 0.910 to 0.911). In conclusion, micro-RNAs up-regulated in HCCs are detectable in plasma but have a poor performance as screening biomarkers of HCC.

## 1. Introduction

Globally, hepatocellular carcinoma (HCC) is the sixth most common form of cancer, and the third leading cause of cancer-related death [1]. Early detection of small tumors enables surgical treatment with curative intention, whereas advanced tumors still carry a poor prognosis despite recent breakthroughs in systemic treatments [2]. Hence, ultrasound surveillance of risk groups for HCC development is advocated by international guidelines [3]. However, ultrasound has its drawbacks, such as investigator dependency and possible limited visibility due to body constitution, resulting in a sensitivity of only 33–61% for the discovery of small tumors [4]. Therefore, plasma biomarkers have attracted great interest as an alternative or complementary approach [5]. Presently, alpha-fetoprotein (AFP), lens culinaris agglutinin-reactive fraction of alpha-fetoprotein (*AFP*-*L3*) and des-gamma-carboxy prothrombin (DCP) have been most extensively investigated [6]. Unfortunately, they all have a poor sensitivity by themselves, although in combination with sex and age the results are more promising [7]. Nevertheless, there is still an urgent need to find additional plasma biomarkers for the early detection of HCC.

In this context, focus has been directed towards the circulating non-coding RNA fractions named microRNAs (miRNAs) as potential biomarkers for HCC [8]. MicroRNAs are small RNA molecules typically numbering 20–23 base pairs [9]. Approximately 2000 different miRNAs in humans have been described. In animal cells, most miRNAs exert post-transcriptional gene regulation by binding to their target mRNA. The mRNA is usually down-regulated through translational suppression, mRNA deadenylation or mRNA decapping, the latter two influencing their stability [10]. In physiological and also pathological conditions (such as cell injury or cell death), miRNAs are released or secreted in extracellular vesicles such as exosomes [11]. Circulating miRNAs can originate from the tumor itself or be derived from other tissues where altered plasma levels in patients with HCC or cirrhosis could be either of hepatic or of extrahepatic origin [12].

Over recent years, a vast body of literature has explored the performance of various miRNAs as diagnostic biomarkers of HCC. Most are exploratory, cross-sectional studies with varying control groups, such as healthy controls, non-cirrhotic hepatitis patients, or patients with cirrhosis [8,11,13,14,15,16,17,18,19,20,21,22,23,24,25]. The results are divergent, and many of the proposed miRNAs are described only in one or a few scientific reports. Furthermore, many miRNAs may be both up- and down-regulated in HCC patients, which could lead to analytical difficulties in clinical practice. However, a small number are reported to be up-regulated in HCC patients in more than a few publications, the most common of which are miR-21 [11,15,23,26,27,28], miR-29 [22,26,29,30,31], miR-192 [11,15,30], miR-199 [15,31,32], miR-221 [26,33,34], miR-222 [26,34], and miR-224 [18,32,35]. Of four meta-analyses that evaluated the diagnostic value of miRNAs for HCC detection, three cautiously encouraged their role as biomarkers [27,36,37], whereas one concluded that adding miRNA to the protein biomarkers AFP or DCP did not improve the diagnostic performance [28].

In the present study, we hypothesized that miRNAs would be up-regulated in tumor tissue from HCCs, compared to their corresponding levels in the surrounding liver parenchyma, and that they could be detected in plasma. Furthermore, we aimed to determine if these miRNAs could discriminate HCC patients from those with chronic liver disease or cirrhosis, i.e., the primary target group for HCC surveillance. First, we determined the miRNA expression in HCCs and surrounding liver tissue from resected livers, and the most differentially elevated miRNAs in HCCs (in comparison to surrounding tissue) with an adequate level of expression were then analyzed in corresponding plasma from the same patients. Finally, we compared the levels in a separate cohort of patients with either HCC or cirrhosis.

## 2. Results

### 2.1. Overview of the Cohorts

**Figure 1:**
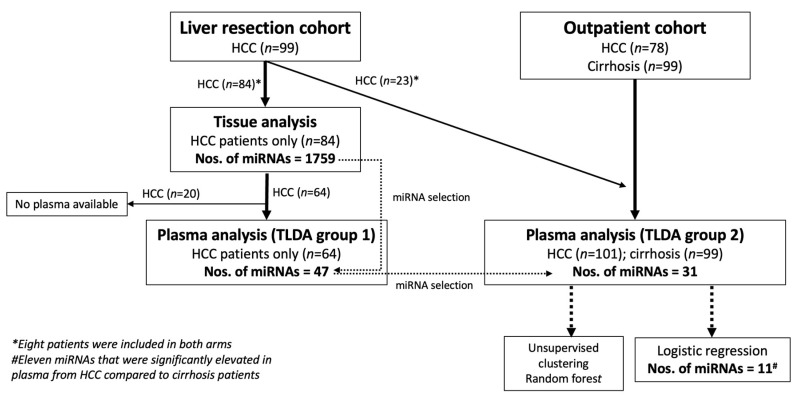
Overview of patient flow in the project. The Liver resection cohort included 99 HCC, while the Outpatient cohort included 78 HCC and 99 cirrhosis. Analysis of miRNA was first performed in liver tissue and plasma from patients in Liver resection cohort, with the results from liver tissue dictating which miRNA to be analyzed in plasma (TLDA group 1). Plasma miRNAs were then analyzed in a cohort with all of the patients in the Outpatient cohort and 23 HCC patients from the Liver resection cohort (of which 8 patients were already analyzed). Comparing HCC with cirrhosis, 11 miRNAs were found to be significantly different between HCC and cirrhosis.

Altogether, 276 patients were included in the present study, 99 from the liver resection cohort (all with HCC) and 177 from the outpatient cohort (78 with HCC and 99 with cirrhosis without HCC). The patient flow is summarized in Figure 1. In the first step, both HCC and surrounding non-tumorous liver tissue were analyzed with RNAseq in 84 patients from the liver resection cohort. In the second step, 64 of these patients had plasma available for miRNA analysis and were analyzed in the first TLDA run. In the final step, patients from the outpatient cohort (*n* = 177), together with 23 patients from the liver resection cohort (of whom 15 patients were not previously analyzed, and 8 patients were previously analyzed with RNAseq on tissue) were selected for the second TLDA run (*n* = 200).

The clinical characteristics of the 200 patients with HCC or cirrhosis analyzed in the second TLDA run are demonstrated in Table 1. The HCC patients were older (70.2 vs. 63.8 years, *p* < 0.01), with a higher proportion of males (83% vs. 66%, *p* < 0.01), and with less underlying alcohol-related liver disease as compared to those with cirrhosis only. Among the HCC patients, 70% had cirrhosis and 30% had a non-cirrhotic or fibrotic liver.

### 2.2. RNAseq

A total of 1759 miRNAs were identified in HCC and surrounding tissues obtained from the liver resection cohort. After selection of those miRNAs with a log-2 fold-change above 0.5 and an adjusted *p*-value below 0.05, 197 miRNAs remained. Of these, 40 miRNAs had a normalized read count above 200 and were chosen for the first TLDA run on plasma from the patients in the liver resection cohort.

The endogenous miR-16-5p used for normalization in the TLDA analysis was analyzed in HCC and surrounding liver tissue. The absolute fold-change was 1.013 (not significant).

### 2.3. First TLDA Run

The run was performed in triplicate on a 48-miRNA chip format, giving space for 47 miRNAs, in addition to miR-16-5p as the endogenous control. Apart from the 40 miRNAs selected from the RNAseq, another 7 miRNAs from the literature were added as comparators (see Table 2).

The results from the triplicate were averaged and sorted in decreasing CT-values. The 24 miRNAs with the lowest CT-values (i.e., highest expression) in the first TLDA run were subsequently chosen for analysis in the second TLDA run.

### 2.4. Second TLDA Run

The second TLDA run was performed in triplicate on a 32-miRNA chip format giving space for 31 miRNAs in addition to miR-16-5p as the endogenous control. In this run, we selected the 24 miRNAs with the lowest CT value from the first TLDA run and added seven additional miRNAs from literature as comparators (Table 3). Eleven miRNAs were significantly elevated (*p* < 0.05) in plasma from patients with HCC as compared to cirrhosis. MiR-122 was elevated in plasma from HCC patients, but without statistical significance. Plasma levels of miR-21 did not show any difference between HCC and cirrhosis patients.

### 2.5. Principal Component Analysis (PCA)

PCA-analysis performed on plasma expression levels of miRNAs in the second TLDA analysis (*n* = 31) demonstrated that the principal component 1 explained 24.2% of the variation, and the principal component 2 explained 16.2%. Visual analysis of the PCA graph could not demonstrate any clear clustering between the HCC group and the cirrhosis group (Supplementary Figure S1).

### 2.6. Hierarchical Clustering

Hierarchical clustering performed on plasma expression levels of the miRNAs in the second TLDA analysis (*n* = 31) divided the cohort in two or three major clusters. Visually, none of the clusters showed a clear overweight of either HCC or cirrhosis patients (Supplementary Figure S2).

### 2.7. Logistic Regression

Stepwise logistic regression, using the bi-directional algorithm for the 11 significantly elevated miRNAs in HCC patients (Table 3), demonstrated only miR-93-5p and miR-151a-3p to be significantly associated with HCC, with a combined AUC of 0.662.

### 2.8. Random Forest

The random forest model had an out of bag estimated error rate of 38%, thus, only 62% of the tested samples were correctly classified. The AUC of the model including 31 miRNAs and 500 trees was calculated to be 0.786.

### 2.9. Combination with AFP and DCP

Plasma was available for extended analysis of AFP and DCP from 143 of the 200 patients in the second TLDA group. In this sub-cohort, the combination of AFP and DCP yielded an AUC of 0.816. Adding miR-93-5p and miR-151a-3p to AFP and DCP increased the AUC to 0.832. However, when also including sex and age, the addition of miR-93-5p and miR-151a-3p did not improve the AUC further (from 0.910 to 0.911, see Table 3 and Supplementary Figure S3).

### 2.10. MiRNA Levels in Plasma from HCC Patients with and without Cirrhosis

Expression of miR-93-5p and miR-151a-3p in plasma from patients with HCC and cirrhosis were similar to those observed in HCC patients without cirrhosis (*p* = 0.26, and *p* = 0.90, respectively).

### 2.11. MiRNA Levels in Plasma Excluding BCLC C and D

In the second TLDA run, results of the 31 single miRNA AUCs excluding HCC-patients with BCLC C or D (*n* = 26) showed a median AUC of 0.546, as compared to a median AUC of 0.552 when all HCCs were included (*p* = 0.05). The combination of miR-93-5p and miR-151a-3p increased from AUC 0.662 to 0.665 when BCLC C and D were excluded (not significant).

### 2.12. MiRNA Levels in Plasma Excluding miR-16-5p Outliers

In the second TLDA run, a total of 7 patients (3 HCC, 4 cirrhosis) had normalized miR-16-5p above 1.5 times IQR in each group, respectively. The median AUC of 31 miRNAs after exclusion of outliers were 0.564, in comparison to 0.552 with all patients included (*p* < 0.01). The combination of miR-93-5p and miR-151a-3p decreased from AUC 0.662 to 0.660 after exclusion of the miR16-5p outliers.

## 3. Discussion

In this study, we investigated whether circulating tumor-associated miRNAs could serve as potential new biomarkers for HCC. In our initial RNAseq analysis, we found several miRNAs that were elevated in HCC tissue compared to surrounding non-tumorous tissue. Our first analysis with TLDA determined which of these had the highest levels in plasma from the same patients. The expressions of the miRNAs with the highest plasma levels were then determined in a largely new cohort of patients with HCC or cirrhosis, and multivariable models were constructed.

Of the 31 miRNAs analyzed in the second TLDA cohort, only eleven were significantly elevated in plasma from HCC patients when compared to the patients with cirrhosis. However, none of the eleven miRNAs alone had an AUC above 0.67. Thus, we conclude that no single miRNA in the present study possessed enough precision to qualify as a plasma biomarker to distinguish HCC from cirrhosis.

Our results are in contrast with the literature, where several miRNAs have been evaluated and found associated with HCC, although the results are very diverse. A literature search on PubMed (keywords “mir”, “hcc”, “diagnostic” and “biomarker”) found over 700 publications from 2015 to 2023, with more than 350 different miRNAs described to be associated with HCC. More than half of the reported miRNAs are only mentioned in one article. Also, while some studies suggest a positive association between a specific miRNA and HCC, others indicate the opposite for the same miRNA. This suggests that the levels of miRNAs associated with HCC might not be consistent between patients, reducing the usefulness of miRNA in the diagnosis of HCC.

In the literature, miR-122 and miR-21 are commonly associated with HCC. MiR-122 is the most abundant miRNA in the liver. It is involved in several liver functions, both physiological and pathological, such as differentiation of hepatocytes, lipid metabolism, and tumor suppression [38]. Previous studies have suggested that miR-122 is commonly increased in patients with HCC [39]. In our cohort, there was a non-significant trend (*p* = 0.07) towards increased miR-122 in plasma from HCC patients compared to those with cirrhosis.

MiR-21 is the second most cited miRNA associated with HCC. This miRNA is involved in embryonal development, cardiovascular diseases and inflammation [40]. In the liver, miR-21 has been associated with viral hepatitis, metabolic dysfunction-associated steatotic liver disease, alcohol-related liver disease and HCC [41]. In most studies, miR-21 is described to be increased in HCC [42], but some publications have shown the opposite [43]. We found similar miR-21 plasma levels in patients with HCC compared to cirrhosis, strengthening the assumption that miR-21 is an unreliable plasma biomarker for HCC.

Although single miRNA showed poor ability to distinguish HCC from cirrhosis, combinations of several miRNAs might still improve the results. In our study, stepwise logistic regression, random forest, principal component analysis (PCA) and hierarchical clustering were used to evaluate the performance of various miRNA combinations.

Stepwise logistic regression showed miR-93-5p and miR-151a-3p to be significantly associated with HCC. Both miRNAs were elevated in HCC tissue as compared to surrounding liver in the resection cohort. Furthermore, they were detected in plasma from the same patients, and in the largely independent TLDA 2 group they were significantly increased in plasma from HCC patients as compared to those with cirrhosis. This would imply that they originate from the tumor tissue itself and could act as potential HCC plasma biomarkers. However, their combination only yielded an AUC of 0.662, while an increase in AUC from 0.816 to 0.832 could be seen if they were added to AFP and DCP. This improvement disappeared when sex and age were also included in the algorithm (Table 4).

MiR-93 has previously been shown to promote HCC invasion and metastasis [44], and its down-regulation had an opposite effect [45]. In line with our results, Xue et al. [46] found elevated miR-93 in the circulation of HCC patients, although not evaluated in combination with AFP and DCP. On the other hand, to the best of our knowledge, miR-151a has not been associated with HCC, but with toxic liver damage and obesity [47,48], and other diseases like atopic dermatitis [49] and glioblastoma [50].

The random forest algorithm was used as a method to find patterns in the miRNAs that could distinguish HCC from cirrhosis. Using the results from 31 miRNAs and 500 trees, the detection rate was only 62% (with an AUC of 0.786). Although being better than the regression model with miR-93-5p and miR-151a-3p, the random forest model did not provide any improvement when compared to the regression model containing only AFP and DCP.

In addition, we employed unsupervised models to find patterns, regardless of the grouping variables. The dataset was investigated both with PCA and hierarchical clustering, but neither method could define any clusters that could separate patients with HCC from those with cirrhosis.

We conclude that no combination of miRNAs, neither by themselves nor together with AFP, DCP, sex and age, can improve the performance as HCC plasma biomarkers in cirrhotic patients. The results from unsupervised methods supported this conclusion.

To investigate if different stages of HCC would influence the miRNA expression, we performed a subgroup analysis in which BCLC C and D were excluded. The median AUC of all 31 miRNAs was slightly reduced (from 0.552 to 0.546, *p* = 0.05), thus with a trend towards significance but with limited clinical relevance. Likewise, the slightly better AUC of the combination of miR-93-5p and miR-151a-3p after exclusion of the advanced HCCs (from 0.662 to 0.665) was not significant and does not change the conclusions from the study.

In contrast to our conclusion, there are previously proposed scoring systems in the literature that combine miRNAs with protein biomarkers. For instance, Fang et al. [51] suggested that a combination of miR-16, miR-122 and the protein alpha-fetoprotein could increase AUC to 0.862. On the other hand, and in concordance with our results, Malik et al. [28] showed that adding miRNAs to the protein biomarkers AFP or DCP did not improve the diagnostic performance further. These results reflect the diverging roles of miRNAs in the diagnosis of HCC, indicating that further research is needed.

The importance of choosing the optimal normalization strategy is crucial in all miRNA studies. Endogenous controls utilizing housekeeping miRNAs are easy to implement and can adjust for both analytical and biological factors. However, the choice of normalization miRNA is essential. We decided to use the commonly utilized miR-16 as the normalization miRNA in this study. While one study suggests an association between miR-16 and HCC [51], making miR-16 questionable to use as normalization in HCC-studies, other studies show the opposite [52]. Also, in our tissue data, miR-16 had similar expression levels in HCC as in surrounding tissue, suggesting that miR-16 is not associated with HCC. Since no consensus is established regarding which miRNA to use, we considered miR-16 as an acceptable normalization miRNA in the present study.

The main strength of our study is the systematic and multi-step approach to find tumor-specific miRNA candidates that could be circulating biomarkers for HCC. Starting from HCC tissue, we are confident that the findings in circulation would have an increased chance of being of pathophysiological relevance. Other studies, focusing only on circulating miRNAs, cannot reasonably argue that the differences are of hepatic origin rather than circumstantial changes originating from other tissues, in contrast to our investigation where that argument can be made.

Another strength are the analytical methods used which allowed us to screen over 1700 miRNAs. TLDA, but especially RNAseq, can simultaneously measure a wide range of miRNAs. By starting with RNAseq in tumor and surrounding liver tissue and analyzing all known miRNAs, we reduced the risk of missing any relevant tumor-specific miRNAs.

We acknowledge several weaknesses of the study. Firstly, the limited sample size of 84 individuals in the RNAseq analysis and 200 in the second plasma analysis must be considered when interpreting the results. A limited sample size reduces the statistical power and may affect the external validity of the study. Since this study focuses on the main target group for HCC surveillance (i.e., patients with liver cirrhosis), the results cannot automatically be generalized to non-cirrhotic patients (for example non-cirrhotic HBV). The study also lacks a validation cohort. However, the overall low AUC values obtained in the miRNA expression analyses and the large overlap of miRNA expressions in plasma between HCC and cirrhosis patients suggest that even an increased sample size, or a validation cohort, would likely generate a similar negative result.

Secondly, no subgroup analysis was performed on the basis of disease etiology. The limited sample size would render any subgroup too small to be reliable in this study. Thus, we cannot rule out that there are specific subgroups in which miRNA could play a role for HCC diagnosis.

Thirdly, thirty percent of the HCC patients were non-cirrhotic, reflecting the varied panorama of HCC patients undergoing liver resection. Thus, the cirrhosis prevalence differed between the groups. However, we believe that the use of paired samples (tumor and surrounding) from the same patients in the resection cohort could partly adjust for the presence of cirrhosis or not, since either both or neither of the paired samples were from a cirrhotic liver. Furthermore, we compared the plasma levels of miR-93 and miR-151a in patients with HCC both with and without cirrhosis, and there were no significant differences between these groups.

Fourthly, we could not rule out an effect of hemolysis on miR-16 in a few of the samples. Absorbance measurements at 414 nm were not carried out, and in a total of seven samples miR-16 was elevated more than 1.5 times IQR. However, the seven samples were evenly distributed between the groups (3 HCC, 4 cirrhosis) and after excluding these seven cases the median AUC of 31 miRNAs increased somewhat from 0.552 to 0.564, and the combination of miR-93-5p and miR-151a-3p decreased from AUC 0.662 to 0.660. Thus, we assess that hemolysis would not have a significant impact on the results.

Lastly, the focus of this study was differentially expressed miRNAs between HCC and the surrounding non-tumorous tissue. Any global miRNA changes in the whole liver due to HCC would therefore not be detected. To partly address this, we added 14 additional miRNAs which have been described as associated with HCC in the literature to the two TLDA analyses, three of which were significantly elevated in plasma from HCC as compared to plasma from patient with cirrhosis.

In summary, although several miRNAs displayed significantly different expression levels in plasma from patients with HCC compared to those with cirrhosis, no single miRNA had sufficient performance to discriminate between these two groups. In logistic regression analysis, miR-93-5p and miR-151a-3p in plasma were significantly associated with HCC as compared to cirrhosis. However, as an HCC biomarker, this miRNA combination yielded a suboptimal performance. Adding these two miRNAs to AFP and DCP tended to increase the diagnostic performance, however; this increase disappeared when sex and age were also included in the analysis. In conclusion, up-regulated tumor-associated miRNAs detectable in plasma have an insufficient performance as diagnostic biomarkers of HCC. As of today, miRNAs, neither as single biomarkers nor part of a scoring system, can rival their protein-based counterparts.

## 4. Methods

### 4.1. Patients

Patients were prospectively included at the Department of Upper Gastrointestinal Diseases at Karolinska University Hospital, from 6 April 2011, to 9 March 2020. Inclusion criteria were signed informed consent; age > 18 years; patients undergoing liver resection for HCC (the “liver resection cohort”); alternatively outpatients at the clinic, either with liver cirrhosis undergoing surveillance or a newly diagnosed and untreated HCC (the “outpatient cohort”), see Figure 1. The study was approved by The Swedish Ethical Review Authority (EPM 2021-06132-02) and conducted according to the Declaration of Helsinki and Istanbul.

HCCs were diagnosed by radiology (European Association for the Study of the Liver criteria in a cirrhotic liver) or histology (liver biopsy or liver resection specimen). Liver cirrhosis was diagnosed either by (a) having a transient elastography (Fibroscan) value above 15 kPa in combination with an underlying liver disease, or (b) a liver biopsy demonstrating liver fibrosis stage 4, or (c) having clinical and radiological signs of portal hypertension in combination with an underlying liver disease.

Clinical data obtained from the medical records included age, sex, presence of cirrhosis, Child–Pugh score, model of end-stage liver disease (MELD) score, liver disease etiology, platelet count, bilirubin, albumin, prothrombin time international normalized ratio (PT-INR), ascites, tumor size, tumor number, macrovascular invasion (MVI), extrahepatic spread (EHS), BCLC group and Milan stage.

### 4.2. Sampling

From the operating room, resected liver specimens were immediately transferred to the pathology department. After initial macroscopic evaluation, tissue samples from both the HCC and surrounding non-tumorous parts were excised and snap-frozen at −80 degrees Celsius until analysis.

Plasma samples were obtained by venipuncture, using standard procedures, into tubes containing ethylenediaminetetraacetic acid (EDTA). Plasma was separated after centrifugation at 2000× *g* for 10 min and promptly frozen at −80 degrees Celsius until further analysis.

### 4.3. RNA Extraction from Tissue

From the tissues obtained from the liver resection group, total RNA was extracted from both HCCs and the surrounding non-tumorous liver tissue using lysis tubes (Precellys, Bretonneux, France) and QIAzol Lysis Reagent (Qiagen, Hilden, Germany). Using RNeasy Mini MinElute kits (Qiagen, Hilden, Germany) according to the manufacturer’s protocol, total RNA was purified and eluted in 14 µL of RNase-free water to be used for miRNA analysis. RNA integrity was evaluated with an Agilent Bioanalyzer (Santa Clara, CA, United States), and samples with RNA integrity number (RIN) < 4 were excluded.

### 4.4. RNA Extraction from Plasma and cDNA Synthesis

To eliminate any residual platelets, 250 µL of plasma were centrifuged at 2000× *g* for 10 min, and 200 µL of the supernatant was retrieved. RNeasy Mini kits (Qiagen, Hilden, Germany) were used to extract total RNA from each sample. cDNA synthesis was then performed using TaqMan advanced cDNA synthesis kit (Thermo Fisher, Waltham, MA, USA) according to the manufacturer’s instructions. 

### 4.5. RNAseq on Tissue RNA

Library preparation and RNAseq were performed on extracted tissue RNA using TruSeq protocols from Illumina. RNAseq was subsequently performed on an Illumina HiSeq 2500 platform at the Swedish National Genomics Infrastructure core facility (Uppsala, Sweden). All steps were performed according to standard protocols from Illumina. An average read yield of 25.4 million reads per sample was obtained with an average >Q30 quality of 96.2%. Trimmed reads were iteratively aligned to miRbase v21, allowing for first zero and then one mismatch with BowTie2, and separately to the GRCh37 reference genome. An average of 32.2% of reads could be aligned to mature miRNAs, and an additional 38.2% to precursor miRNAs.

Counts were normalized using the trimmed mean of M-values (TMM) method before further analysis. High quality data was obtained for 84 tumor/adjacent non-tumorous sample pairs.

For each miRNA in the normalized data, the fold-change between HCC tissue and surrounding non-tumorous tissue was calculated together with a *p*-value from a paired *t*-test, adjusted for multiple testing by the Benjamini–Hochberg method, using Python scripts. The median log-2 fold-change and *p*-values were then used to rank the miRNAs.

### 4.6. First TLDA Run (TLDA Group 1)

The miRNAs with the highest fold-change between HCC tissue and surrounding non-tumorous liver tissue were analyzed in plasma from the same HCC-patients that had undergone RNAseq (the “liver resection cohort”), see Figure 1. Custom made TaqMan Low Density Array (TLDA) kits from Thermo Fisher (Waltham, MA, USA) were used to analyze the miRNAs in triplicate, according to the manufacturer’s protocol. Results from triplicates were averaged and CT-values were normalized against the endogenous miR-16-5p.

### 4.7. Second TLDA Run (TLDA Group 2)

The miRNAs with the highest expression in TLDA group 1 were re-analyzed on plasma from HCC and cirrhosis patients in a largely new cohort (TLDA group 2), see Figure 1, using the same TLDA platform. Triplicates were again averaged and resulting CT-values were normalized against the endogenous miR-16-5p.

### 4.8. AFP and DCP

Alpha-fetoprotein (AFP) and des-gamma-carboxy prothrombin (DCP) were analyzed on the HCC and cirrhosis patients in the TLDA group 2 on a Roche Cobas 8000 e601 module using electrochemiluminescence according to the manufacturer’s instructions.

### 4.9. Statistics

For descriptive analysis, the median and interquartile range were used for continuous variables, and total number and percentage were used for categorical variables. Wilcoxon’s test was used to compare continuous variables and Chi-2 test for categorical variables.

The minimum sample size was calculated by assuming a 25% difference between groups with a coefficient of variation of 50%, to be approximately 65 patients in each group.

### 4.10. Unsupervised Models—PCA and Hierarchical Clustering

The unsupervised models, based on principal component analysis (PCA) and hierarchical clustering, were used to find patterns or clusters in the TLDA group 2 dataset to evaluate a possible association of miRNAs to HCC or cirrhosis.

PCA was used to reduce the number of dimensions and plotting all samples on a 2D graph (principal component 1 and 2) in order to visualize occurrence of clusters based on miRNA expression in plasma.

Hierarchical clustering, by clustering similar samples together depicted as a dendrogram, was also used to divide the TLDA group 2 into separate clusters and evaluate the occurrence of HCC and cirrhosis within each group.

### 4.11. Supervised Models—Logistic Regression

For the multivariable models, stepwise logistic regression was used. All significantly elevated miRNAs in single miRNA comparisons between HCC and cirrhosis patients in the TLDA group 2 (using Wilcoxon’s test) were evaluated. The resulting significant miRNAs were then tested in combination with AFP and DCP, with and without sex and age.

### 4.12. Supervised Models—Random Forest

Lastly, a random forest algorithm was applied to miRNA in plasma (*n* = 31) in TLDA group 2 to further evaluate the discriminatory power of miRNAs in distinguishing HCC from cirrhosis. Random forest combines the prediction power from multiple randomly generated decisions trees to evaluate if there is any discriminatory power in the investigated miRNAs. The random forest model was run using 500 trees and five variables tried at each split.

All statistical calculations, unless stated elsewhere, were made in R and R-studio (v 1.4). *p* < 0.05 was considered to be statistically significant.

### 4.13. Sensitivity Analyses

To evaluate if tumor stage would affect miRNA expression, a sensitivity analysis was performed in which the AUCs of miRNAs were calculated after exclusion of Barcelona Clinic Liver Cancer (BCLC) stage C and D patients.

A sensitivity analysis was also performed to evaluate if hemolysis would have had impact on the normalization with miR-16-5p. For this purpose, miR-16-5p levels underwent global normalization against the other 31 miRNAs. Elevated miR-16-5p, with values above 1.5 times IQR, were considered as outliers in which hemolysis could not be ruled out. The outliers were excluded and the AUCs of miRNAs were calculated as a sensitivity analysis.

## Figures and Tables

**Table 1:** The clinical characteristics of patients with HCC (*n* = 101) or cirrhosis (*n* = 99), who were analyzed for miRNA in plasma in the second TLDA run (TLDA group 2).

	HCC (*n* = 101)		Cirrhosis (*n* = 99)		*p*-Value
	Median/Number (%)	IQR	Median/Number (%)	IQR	
Age (years)	70.2	65.7–75.0	63.8	56.3–70.4	<0.01
Sex (male)	84 (83%)		65 (66%)		<0.01
Cirrhosis	71 (70%)		99 (100%)		<0.01
Child Pugh					
- A (or no cirrhosis)	70 (69%)		53 (54%)		0.07
- B	24 (24%)		34 (34%)		
- C	7 (7%)		12 (12%)		
MELD score	9	7–11	9	7–12.5	0.23
Etiology					
- No liver disease	14 (14%)		0 (0%)		<0.01
- Viral hepatitis	27 (27%)		18 (18%)		
- Alcohol	29 (29%)		42 (42%)		
- MASLD	23 (23%)		17 (17%)		
- Autoimmune	0 (0%)		9 (9%)		
- Other	8 (8%)		13 (13%)		
Platelets (10^9^/L)	178	117.8–243	141	92.5–193.5	<0.01
Bilirubin (µmol/L)	10.5	7.75–16.25	17	10–26	<0.01
Albumin (g/L)	34	31–36.25	33	29–37	0.76
PT-INR	1.1	1.0–1.3	1.2	1.1–1.4	0.03
Ascites					
- None	77 (76%)		52 (53%)		<0.01
- Mild/moderate	17 (17%)		33 (33%)		
- Severe	7 (7%)		14 (14%)		
Total tumor size (cm)	5.5	3–9.25	-	-	
Number of tumors	1	1–2	-	-	
EHS	11 (11%)		-		
MVI	16 (16%)		-		
BCLC					
- 0, A or B	75 (74%)		-		
- C or D	26 (26%)		-		

EHS = extrahepatic spread. IQR = interquartile range. MASLD = metabolic dysfunction-associated steatotic liver disease. MELD = model of end-stage liver disease. MVI = macrovascular invasion. PT-INR = prothrombin time international normalized ratio.

**Table 2:** Overview of miRNAs analyzed in the first TLDA run (TLDA group 1), including miRNAs found to be elevated in HCC tissue (*n* = 40) and comparators from literature (*n* = 7). CT values are normalized against endogenous miR-16-5p. From these, the 24 miRNAs (depicted with ^†^) with the lowest CT-values were chosen for the second TLDA run.

Micro-RNA in First TLDA Run			
Up-regulated in HCC Tissue (CT-Value)		Comparator from Literature (CT-Value)	
hsa-miR-21-5p ^†^	−0.8	hsa-miR-15b ^†^	0.0
hsa-miR-15a-5p ^†^	−1.0	hsa-miR-18a ^†^	−4.8
hsa-miR-25-3p ^†^	−1.9	hsa-miR-222-3p ^†^	−5.6
hsa-miR-221-3p ^†^	−2.1	hsa-miR-192 ^†^	−5.9
hsa-miR-30b-5p ^†^	−2.2	hsa-miR-375	−7.3
hsa-miR-151b ^†^	−2.6	hsa-miR-130b-5p	−7.3
hsa-miR-425-5p ^†^	−3.0	hsa-miR-147b	−9.2
hsa-miR-93-5p ^†^	−3.3		
hsa-miR-106b-5p ^†^	−3.6		
hsa-miR-484 ^†^	−3.8		
hsa-miR-151a-3p ^†^	−4.7		
hsa-miR-339-5p ^†^	−5.0		
hsa-miR-32-5p ^†^	−5.1		
hsa-miR-151a-5p ^†^	−5.4		
hsa-miR-33a-5p ^†^	−5.5		
hsa-miR-128-3p ^†^	−5.6		
hsa-miR-130b-3p ^†^	−5.7		
hsa-miR-34a-5p ^†^	−6.0		
hsa-miR-339-3p ^†^	−6.4		
hsa-miR-301a-3p ^†^	−6.6		
hsa-miR-106b-3p	−6.6		
hsa-miR-345-5p	−6.8		
hsa-miR-660-5p	−6.8		
hsa-miR-500a-3p	−6.9		
hsa-miR-421	−7.1		
hsa-miR-182-5p	−7.4		
hsa-miR-95-3p	−7.4		
hsa-miR-532-5p	−7.5		
hsa-miR-96-5p	−7.5		
hsa-miR-301b-3p	−7.6		
hsa-miR-155-5p	−7.7		
hsa-miR-21-3p	−7.7		
hsa-miR-502-3p	−7.8		
hsa-miR-1307-3p	−8.4		
hsa-miR-1285-3p	−9.6		
hsa-miR-452-5p	−9.6		
hsa-miR-501-3p	−11.4		
hsa-miR-183-5p	−11.5		
hsa-miR-10b-5p	−13.1		
hsa-miR-98-5p	−15.4		

**Table 3:** Overview of 31 miRNAs analyzed in the second TLDA run (TLDA group 2), comparing patients with HCC (*n* = 101) and cirrhosis (*n* = 99). All results are normalized against miR-16-5p and converted from CT-values to absolute values, where miR-16-5p was set to a value of 1000. Eleven miRNAs were significantly elevated in plasma from HCC patients as compared to patients with cirrhosis without HCC.

	HCC (*n* = 101)		Cirrhosis (*n* = 99)		Comparison	
Micro-RNA	Median	IQR	Median	IQR	*p*-Value	AUC
Micro-RNA with significantly increased levels in HCC patients compared to patients with cirrhosis						
hsa-miR-93-5p	69.9	(46.1–102.9)	46.6	(34.5–66.2)	<0.01	0.669
hsa-miR-339-5p	9.2	(2.1–28.3)	4	(1.4–8.1)	<0.01	0.643
hsa-miR-130b-3p	14.6	(5.2–49.3)	8.9	(2.8–17.7)	<0.01	0.624
hsa-miR-222-3p ^#^	33.1	(16.2–75.7)	20.7	(13.4–38.1)	<0.01	0.618
hsa-miR-151a-3p	76.8	(23.8–212.4)	35.2	(17.3–77.2)	<0.01	0.617
hsa-miR-151b	444.1	(175.2–1082.2)	265.2	(144.9–602.7)	0.01	0.603
hsa-miR-30b-5p	155	(757.6–333.1)	96.1	(43.8–191)	0.02	0.599
hsa-miR-29a-3p ^†^	55.7	(26.4–159.4)	35.2	(20.4–68.8)	0.02	0.599
hsa-miR-221-3p	422.8	(231.3–853.8)	353.8	(212.7–552.9)	0.03	0.589
hsa-miR-532-5p ^†^	5.6	(2–14.9)	2.8	(1.6–10)	0.03	0.588
hsa-miR-151a-5p	218.5	(60.3–773.8)	144.5	(46.7–324.2)	0.05	0.580
Micro-RNA with no significant differences between HCC and cirrhosis patients						
hsa-miR-122-5p ^†^	247.1	(76.5–1278.1)	146.7	(53.2–555.7)	0.07	0.575
hsa-miR-25-3p	607.9	(375.8–1304.1)	475.7	(307.9–1235)	0.10	0.568
hsa-miR-34a-5p	9.3	(0.1–29.2)	4.6	(0.1–16.3)	0.10	0.567
hsa-miR-339-3p	9.7	(1.6–28)	6.7	(3.9–11.4)	0.12	0.564
hsa-miR-15b-5p ^#^	1000.7	(253.1–2629.9)	710.5	(211–2194.7)	0.20	0.552
hsa-miR-182-5p ^†^	23	(5.2–82.9)	14.7	(3.8–80)	0.29	0.544
hsa-miR-301a-3p	2.8	(0–9.8)	1.8	(0.1–6.5)	0.28	0.544
hsa-miR-1246-5p ^†^	8.1	(1.1–28.4)	4.4	(1.6–15.6)	0.31	0.542
hsa-miR-33a-5p	6.3	(0–24.7)	3.7	(0–21.8)	0.35	0.539
hsa-miR-484	197	(109.7–440.7)	167.7	(104–308)	0.34	0.539
hsa-miR-106b-5p	72.9	(30.1–174.5)	57.4	(26.9–122.8)	0.36	0.538
hsa-miR-425-5p	35.1	(18.4–71.9)	32.4	(19.1–48.2)	0.40	0.535
hsa-miR-192-5p ^#^	45.5	(12–180.2)	54	(25.2–138.7)	0.39	0.535
hsa-miR-21-5p	531.1	(289–1601)	522.3	(319.9–1017.1)	0.40	0.534
hsa-miR-15a-5p	530	(425.4–832.2)	609.6	(456.8–785.1)	0.50	0.528
hsa-miR-18a-5p ^#^	43.1	(11.7–85.1)	32.5	(14.1–60.1)	0.55	0.525
hsa-miR-128-3p	28.6	(10.5–65.1)	27	(14.1–43.9)	0.66	0.518
hsa-miR-375-3p ^†^	87.8	(30.3–323.8)	71.8	(42.1–195.9)	0.70	0.516
hsa-miR-155-5p ^†^	25.9	(4.3–121.6)	20.8	(4.9–85.8)	0.73	0.514
hsa-miR-32-5p	32.5	(6.6–79.5)	35.3	(12.7–62.5)	0.86	0.507

IQR = interquartile range, AUC = area under the curve. ^#^ depicts miRNA from literature added in first TLDA run. ^†^ depicts miRNA from literature added in second TLDA run.

**Table 4:** Multivariable models based on logistic regression. Combination of the two significant miRNAs from stepwise logistic regression, miR-93-5p and miR-151a-3p, resulted in an AUC of 0.662, lower than the combination of AFP and DCP (AUC 0.816). The addition of the miRNAs to AFP and DCP improved the results, but when including sex and age to AFP and DCP, no improvement could be seen when adding the miRNAs.

Parameters	AUC for Parameters	Sensitivity at 90% Specificity	AUC for Parameters + Sex & Age	Sensitivity at 90% Specificity
miR93-5p, miR151a-3p	0.662	20%	0.711	32%
AFP, DCP	0.816	38%	0.910	64%
AFP, DCP, miR93-5p, miR151a-3p	0.832	55%	0.911	67%

AUC = area under the curve. AFP = alpha-fetoprotein. DCP = des-gamma-carboxy prothrombin.

## Data Availability

The datasets generated and/or analyzed during this study are available from the corresponding author on reasonable request.

## Supplementary Materials

The following supporting information can be downloaded at: https://www.mdpi.com/article/10.3390/ijms25042414/s1.

## Abbreviations

AFP	alpha-fetoprotein
BCLC	Barcelona Clinic Liver Cancer staging system
DCP	des-gamma-carboxy prothrombin
EASL	European Association for the Study of the Liver
EDTA	Ethylenediaminetetraacetic acid
EHS	extrahepatic spread
HCC	hepatocellular carcinoma
MASLD	metabolic dysfunction-associated steatotic liver disease
MELD	model of end-stage liver disease
miR or miRNA	micro-RNA
mRNA	messenger-RNA
MVI	macrovascular invasion
PCA	principal component analysis
PT-INR	prothrombin time—international normalized ratio
RIN	RNA integrity number
RISC	RNA-induced silencing complex
RNA	Ribonucleic acid
RNAseq	RNA sequencing
TMM	trimmed mean of M-values
UTR	untranslated region
