# Supplementary material for: MicroRNAs as Plasma Biomarkers of Hepatocellular Carcinoma in Patients with Liver Cirrhosis—A Cross-Sectional Study
Source: Int J Mol Sci. 2024 Feb 19;25(4):2414. doi: 10.3390/ijms25042414 (PMC10888674; doi:10.3390/ijms25042414)
Supplement: Supplementary file 1 [file ijms-25-02414-s001.zip › ijms-2850885-supplementary.pdf]

Supplementary material for

MicroRNAs as plasma biomarkers of hepatocellular carcinoma in patients with liver cirrhosis – a cross-sectional study

Robin Zenlander[1,2,3] (ORCID 0000-0003-0919-5580), MD, PhD-student

Hugh Salter[2], PhD

Stefan Gilg[3], MD, PhD

Gösta Eggertsen[1,3], MD, PhD

Per Stål[3,4] (ORCID 0000-0003-2915-1964), MD, PhD

[1] Department of Clinical Chemistry, Karolinska University Hospital, 141 86 Stockholm, Sweden

[2] Department of Laboratory Medicine, Karolinska Institutet, 141 52 Stockholm, Sweden

[3] Department of Medicine, Huddinge, Karolinska Institutet, 141 86 Stockholm, Sweden

[4] Division of Hepatology, Department of Upper GI Diseases, Karolinska University Hospital, 141 86 Stockholm, Sweden

Keywords: HCC, surveillance, circulating biomarkers, miRNA, proteins

Correspondence and reprint requests:

Robin Zenlander

Phone: +46 (0) 72 582 34 27, E-mail: robin.zenlander@ki.se

Supplementary figure S1

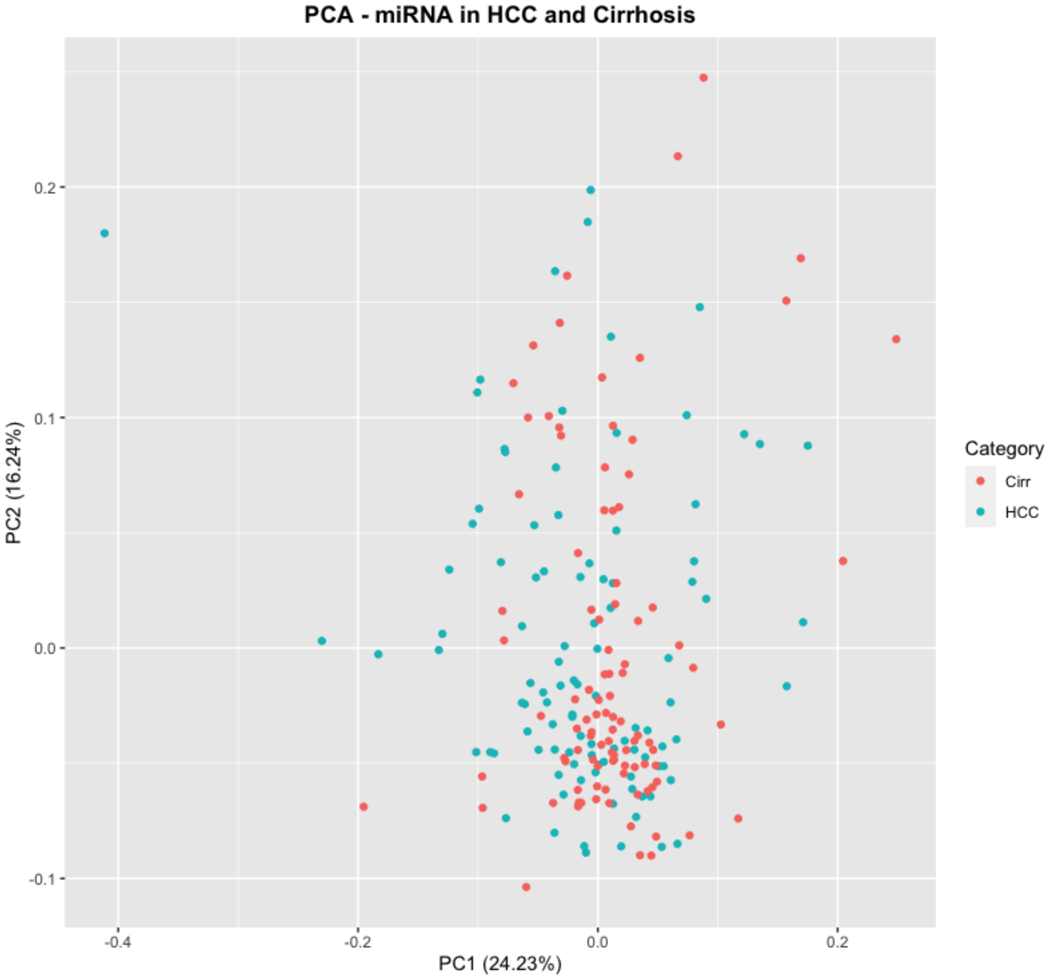

Supplementary figure S1. Principal component analysis (PCA) of microRNA for HCC and Cirrhosis. Principal component 1 (PC1) accounts for 24.23% while principal component 2 (PC2) accounts for 16.24%.

Supplementary figure S2

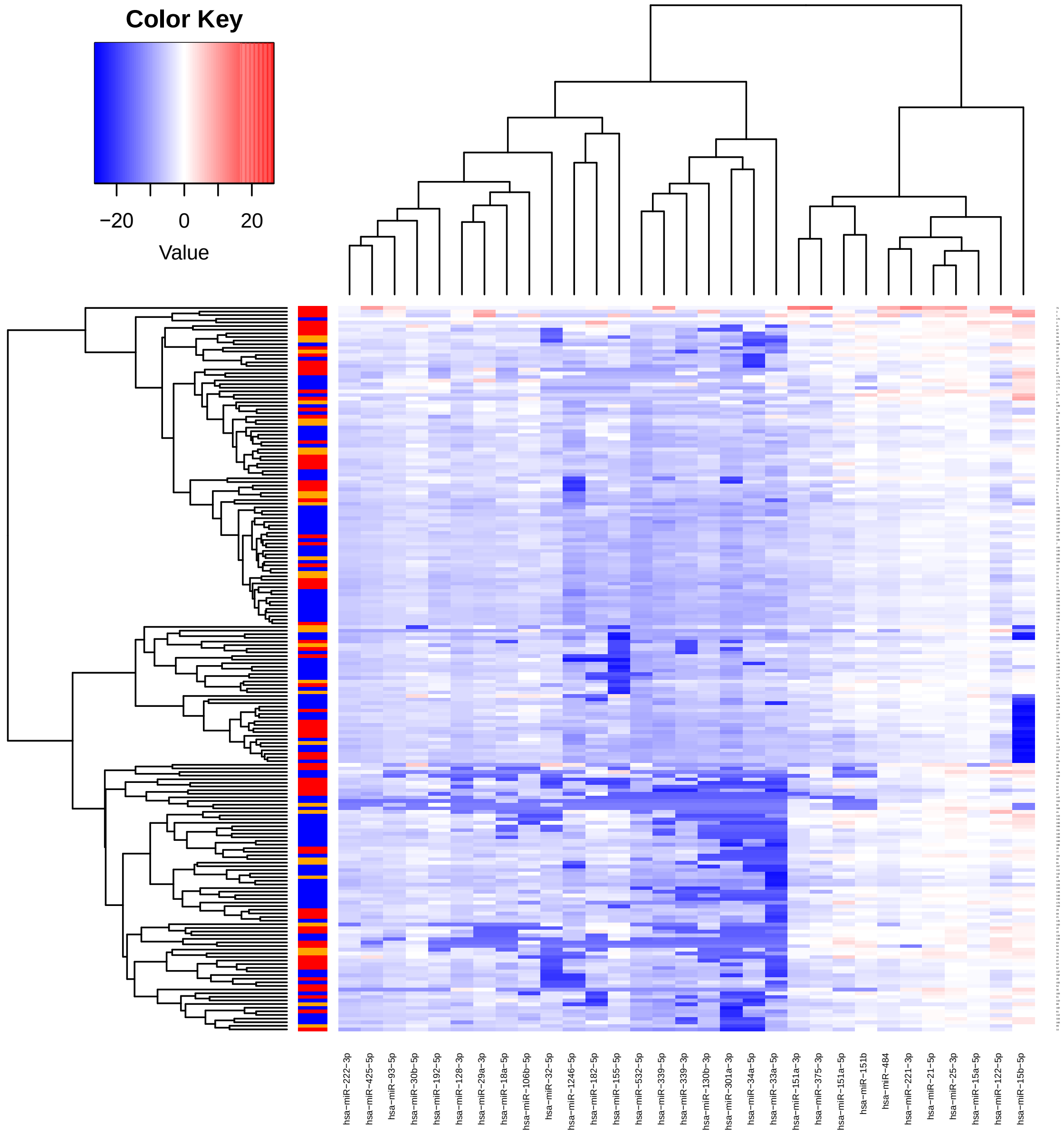

Supplementary figure S2. Hierarchical clustering with patients depicted in rows and microRNA in columns. The Color Key depicts the CT-value for each microRNA. The left row side colors represent patient category with blue = cirrhosis, orange = HCC and red = HCC + cirrhosis. There were no enrichments of either category in the clusters generated.

Supplementary figure S3

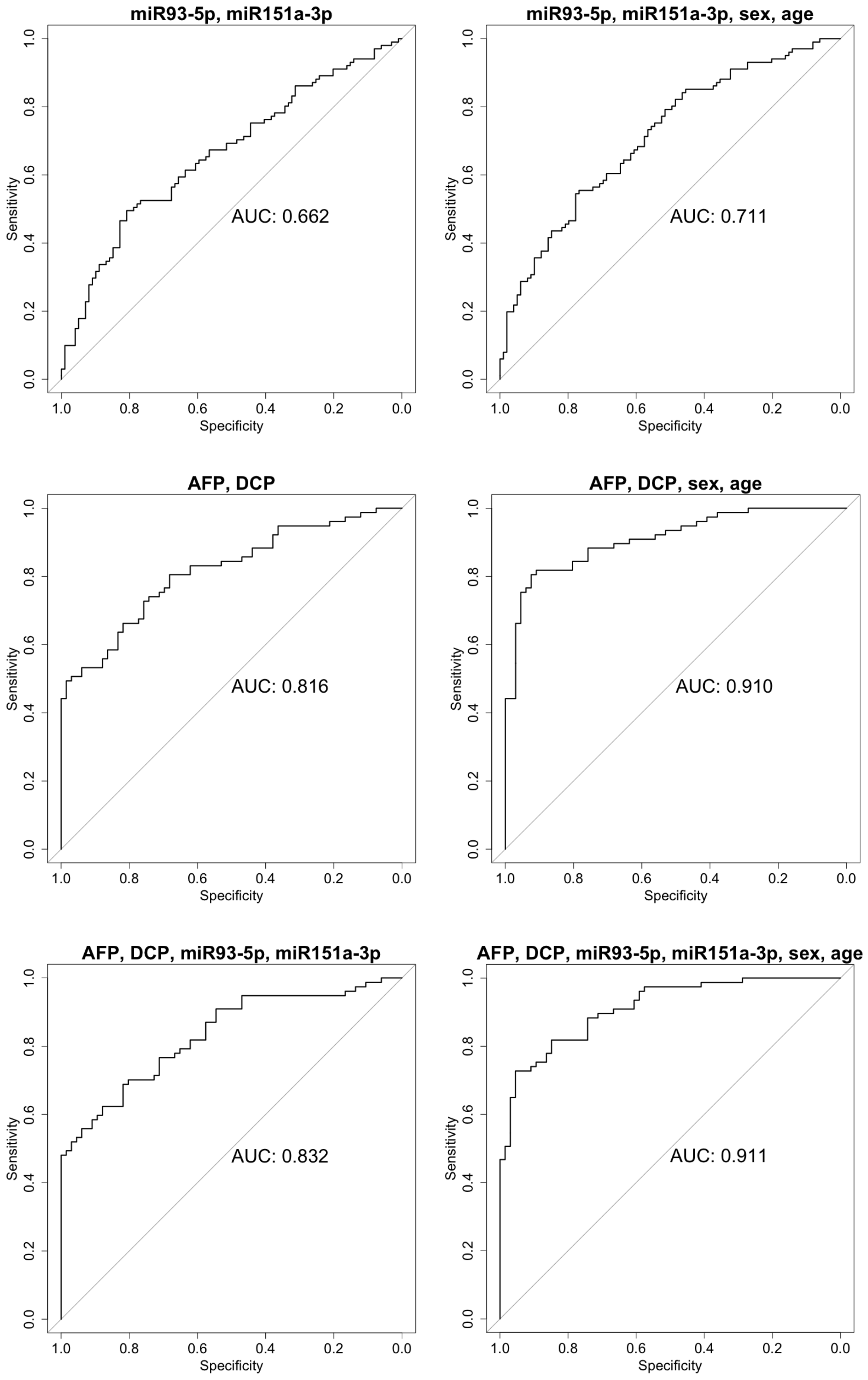

Supplementary figure S3. ROC curves for regression models depicted in Table 4 in main article. AFP and DCP in combination had a AUC of 0.816, but increased to 0.832 when miR93-5p and miR151a-3p were added. However, when sex and age were also included with AFP and DCP, the addition of miR93-5p and miR151a-3p did not improve the AUC further.
